# Cinnamoyl Sucrose Esters as Alpha Glucosidase Inhibitors for the Treatment of Diabetes

**DOI:** 10.3390/molecules26020469

**Published:** 2021-01-18

**Authors:** Surabhi Devaraj, Yew Mun Yip, Parthasarathi Panda, Li Lin Ong, Pooi Wen Kathy Wong, Dawei Zhang, Zaher Judeh

**Affiliations:** 1 School of Chemical and Biomedical Engineering, Nanyang Technological University, 62 Nanyang Drive, N1.2–B1-14, Singapore 637459, Singapore; surabhi.devaraj@gmail.com (S.D.); panda.ju06@gmail.com (P.P.); long008@e.ntu.edu.sg (L.L.O.); pwong003@e.ntu.edu.sg (P.W.K.W.); 2 School of Physical and Mathematical Sciences, Nanyang Technological University, 21 Nanyang Link, Singapore 637371, Singapore; yipy0010@e.ntu.edu.sg (Y.M.Y.); sunstar53@126.com (D.Z.); 3 Institute of Health Technologies, Interdisciplinary Graduate School, Nanyang Technological University, 61 Nanyang Drive, ABN-02b-07, Singapore 637335, Singapore; 4 School of Physics and Engineering, Henan University of Science and Technology, Luoyang 471000, China

**Keywords:** phenylpropanoid sucrose esters, glycosides, natural products, α-glucosidase inhibition, α-amylase inhibition, anti-diabetic

## Abstract

Cinnamoyl sucrose esters (CSEs) were evaluated as AGIs and their enzyme inhibition activity and potency were compared with gold standard acarbose. The inhibition activity of the CSEs against α-glucosidase and α-amylase depended on their structure including the number of the cinnamoyl moieties, their position, and the presence or absence of the acetyl moieties. The inhibitory values of the CSEs **2**–**9** generally increases in the order of mono-cinnamoyl moieties < di-cinnamoyl ≤ tri-cinnamoyl < tetra-cinnamoyl. This trend was supported from both in vitro and in silico results. Both tetra-cinnamoyl CSEs **5** and **9** showed the highest α-glucosidase inhibitory activities of 77 ± 5%, 74 ± 9%, respectively, against acarbose at 27 ± 4%, and highest α-amylase inhibitory activities of 98 ± 2%, 99 ± 1%, respectively, against acarbose at 93 ± 2%. CSEs **3**, **4**, **6**, **7**, **8** showed desired higher inhibition of α-glucosidase than α-amylase suggesting potential for further development as AGIs with reduced side effects. Molecular docking studies on CSEs **5** and **9** attributed the high inhibition of these compounds to multiple π-π interactions and favorable projection of the cinnamoyl moieties (especially O-3 cinnamoyl) in the enzyme pockets. This work proposes CSEs as new AGIs with potentially reduced side effects.

## 1. Introduction

Alpha Glucosidase Inhibitors (AGIs) acarbose, voglibose, and miglitol are effective oral antidiabetic drugs [1]. They function by inhibiting the digestive α-glucosidase and α-amylase enzymes that break down starch and other oligosaccharides. [1] They have a better safety profile compared to other drugs such as insulin, sulphonylureas, biguanides, meglitinides, thiazolidinediones, and SGLT-2 inhibitors [2,3]. However, they are often accompanied by severe gastrointestinal side effects such as flatulence, diarrhea, and bloating, which limit patient acceptance and compliance [4]. The side effects are caused by the fermentation of undigested carbohydrates in the large intestine due to excessive AGIs inhibition of α-amylase [5]. Therefore, it is highly desirable to develop effective AGIs but without these side effects. Such AGIs should have high selectivity causing high inhibition of α-glucosidase and low inhibition of α-amylase.

**Figure 1:**
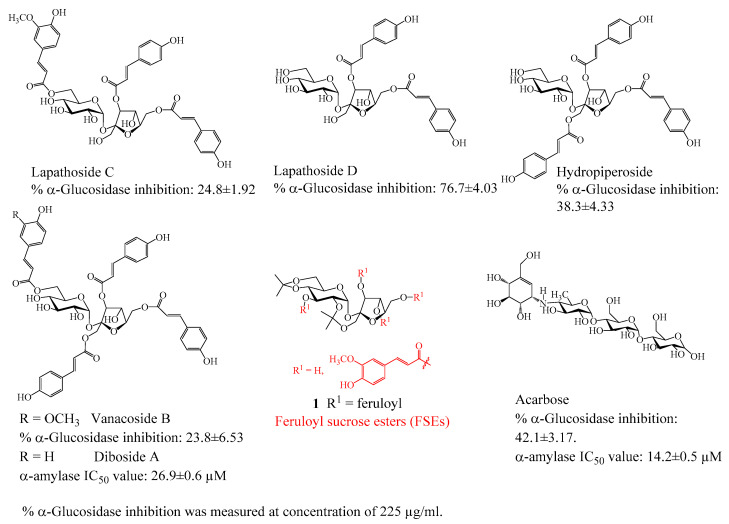
Phenylpropanoid sucrose esters (PSEs) with % α-glucosidase and α-amylase inhibitory activities. Also shown is the general structure of feruloyl sucrose ester (FSE) and acarbose.

Phenylpropanoid sucrose esters (PSEs) are naturally occurring compounds isolated from various plant species such as Arecaceae, Liliaceae, Polygonaceae, Rosaceae, Brassicaceae, and Smilaceae [6]. PSEs feature a sucrose core acylated with one or more phenylpropanoid (trans-PhCH=CH-CO-) moieties such as cinnamoyl, coumaroyl, feruloyl, caffeoyl, and/or sinapoyl moieties [7]. Their extracts were used in various traditional and folk medicines for treating various illnesses [8]. They exhibit a wide range of biological activities including anticancer, antibacterial, antiplasmodial, antioxidant, and acetylcholine esterase inhibitory activities [6,7,9,10,11,12,13,14,15,16,17,18,19]. Additionally, PSEs show variable α-glucosidase and α-amylase inhibitory activities depending on the type, number, and position of the phenylpropanoid moieties on the sucrose core. Examples of these biologically active PSEs include lapathoside C [14], lapathoside D [14], hydropiperoside [14], vanicoside B [14], and diboside A [11] (Figure 1). Several other phenylpropanoid glycosides also showed excellent α-glucosidase and α-amylase inhibition activities [20,21,22,23,24,25].

**Figure 2:**
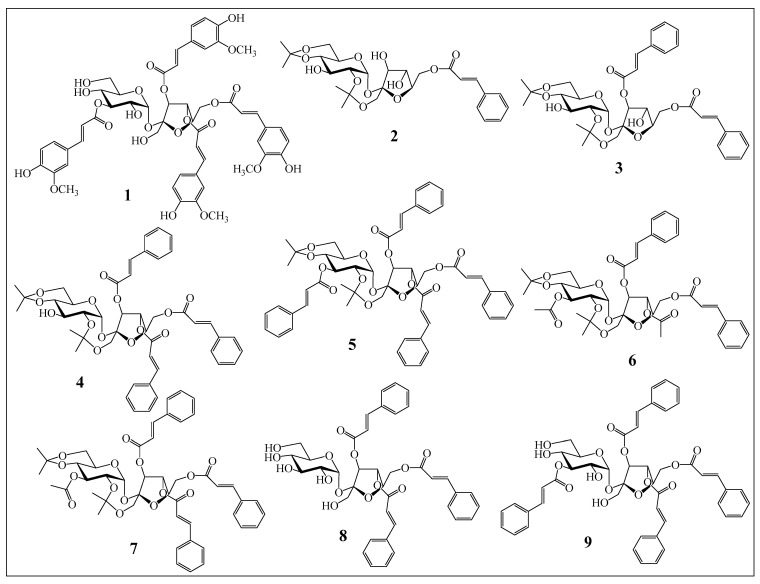
Structures of the investigated cinnamoyl sucrose esters (CSEs) **2**–**9** along with FSE **1**.

We have earlier reported that feruloyl sucrose esters (FSEs) (Figure 1) function as selective AGIs and has the potential to eliminate the side effects associated with the commercial AGIs [26]. FSEs desirably showed higher % inhibition of α-glucosidase and lower inhibition of α-amylase in comparison to acarbose as the AGI gold standard [26]. Specifically, FSE **1** (Figure 2) significantly reduced blood glucose excursion in STZ-treated mice compared to control (starch only) mice. The diisopropylidene bridges, position, and the number of the feruloyl substituents on the sucrose core were key determinants of the selectivity and % inhibition of α-glucosidase and α-amylase [26]. Further research is advantageous to assess other phenylpropanoid moieties beside the feruloyl moieties to obtain lead AGIs candidate for further development.

Herein, we evaluate cinnamoyl sucrose esters (CSEs) as AGIs and compare their % α-glucosidase and α-amylase inhibition activities with gold standard acarbose. The factors that influence the selectivity and inhibition of the enzymes are also discussed.

## 2. Results and Discussions

### 2.1. Design and Synthesis of Cinnamoyl Sucrose Esters ***2**–**9***

**Scheme 1:**
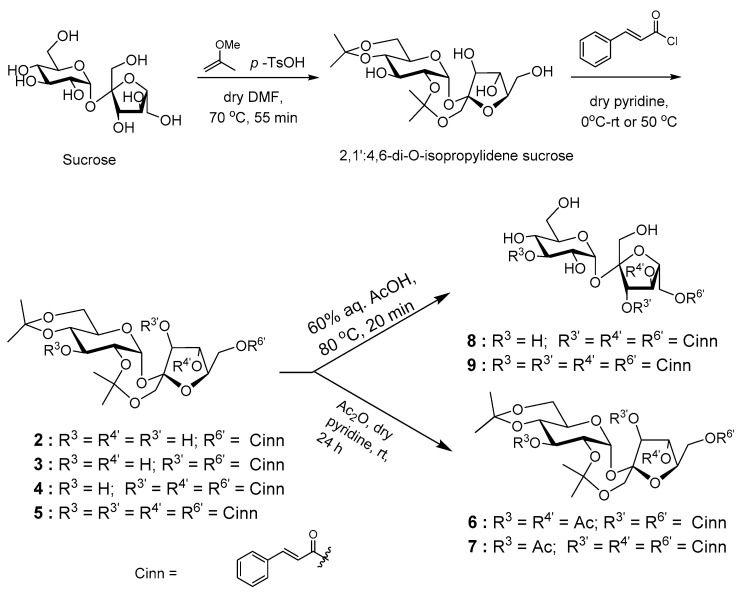
Synthesis of CSEs **2**–**9**.

The inhibition activities of α-glucosidase and α-amylase using natural PSEs [26] and FSEs synthesized in our laboratory [26,27] depended on the structural features of these compounds. Therefore, CSEs **2**–**9** were specifically designed to differ in: (i) the number of cinnamoyl moieties, (ii) the degree of acetylation of the sucrose core, and (iii) the presence or absence of the diisopropylidene bridges on sucrose core (Figure 2). These bridges control the degree of flexibility between the glucose and fructose moieties of the sucrose core. CSEs **2**–**9** were also designed to complement and compare with our inhibition studies using a small library of FSEs such as **1** (Figure 2). CSEs **2**–**9** were synthesized by cinnamoylation of 2,1′:4,6-di-*O*-isopropylidene sucrose with cinnamoyl chlorides in dry pyridine according to our previous work (Scheme 1) [19]. Acetylation of the cinnamoylated compounds was then achieved using acetic anhydride to give the acetylated CSEs [19].

### 2.2. Inhibition of α-Glucosidase and α-Amylase by Cinnamoyl Sucrose Esters ***2***–***9*** In Vitro

**Figure 3:**
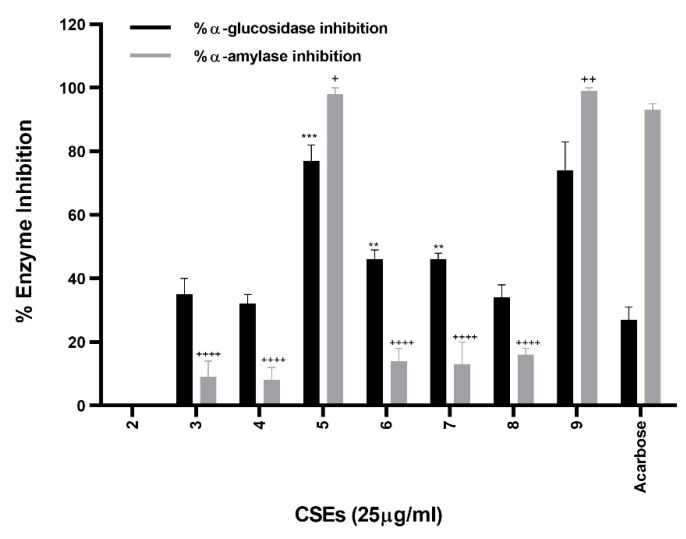
Percentage inhibition of α-glucosidase and α-amylase by CSEs **2–9** with acarbose as the reference standard. Inhibition values are expressed as means ± SD; *n* = 3. CSEs showing less than 5% enzyme inhibition are considered to have No Significant Activity (NSA). ** *p* ≤ 0.01, *** *p* ≤ 0.001, for significant change in α-glucosidase inhibition compared with Acarbose; + *p* ≤ 0.05, ++ *p* ≤ 0.01, ++++ *p* ≤ 0.0001 for significant change in α-amylase inhibition compared with Acarbose.

CSEs **2**–**9** were screened for α-glucosidase and α-amylase inhibition at their maximum solubility limit of 25 μg/mL (Figure 3). Acarbose, the gold standard AGI drug, was used as a reference standard at the same concentration. Except for CSE **2**, all CSEs investigated showed inhibition of both α-glucosidase and α-amylase, and the inhibition was mainly influenced by the number of the cinnamoyl substituents on the sucrose core and to a lesser extent by the acetyl moieties and the diisopropylidene bridges. CSE **2** with one cinnamoyl moiety did not show significant inhibition activity of both enzymes, in agreement with the inhibition results using FSE with one feruloyl moiety. Both CSEs **3** and **4** with diisopropylidene bridges and free OH groups showed similar α-glucosidase inhibition of 35 ± 5% and 32 ± 3%, respectively, and similar α-amylase inhibition activities of 9 ± 5% and 8 ± 4%, respectively (Figure 3). In both cases, the % α-glucosidase inhibition was >4 times higher than the % α-amylase inhibition. An additional cinnamoyl at O-3 as in CSE **5**, caused a huge increase in the % α-glucosidase inhibition to 77 ± 5 and even a bigger increase in the α-amylase inhibition to 98 ± 2%. Again, these results agree with the inhibition trend observed in the case of FSEs where moieties at O-3 lead to higher enzyme inhibition.^26^ Capping of the hydroxyl moieties of CSEs **3** and **4** with acetyl protecting groups to give CSEs **6** and **7** respectively, resulted in a similar increase in both the α-glucosidase inhibition (46 ± 3% and 46 ± 2%, respectively) and α-amylase inhibitions (14 ± 4% and 13 ± 7%, respectively). In both cases, the % α-glucosidase inhibition remained roughly >4 times higher than the % α-amylase inhibition as seen in the case of free OH in CSEs **3** and **4**. The modest increase in selectivity may be attributed to the steric effects that the acetyl groups cause, which favors better docking of CSEs **6** and **7** at the active sites of the enzymes. Though the increase in inhibition was modest, acylation of the OH groups with other larger acetyl groups could be a means to influence the inhibition activities. CSE **8** without the diisopropylidene or acetyl groups exhibited a similar α-glucosidase inhibition value of 34 ± 4% as compared to its counterpart, CSEs **4** with 32 ± 3%. However, it’s α-amylase inhibition value of 16 ± 2% is double that of CSEs **4** at 8 ± 4%. These results once again clearly show that the diisopropylidene bridges on the sucrose core have no significant influence on α-glucosidase inhibition: a similar trend observed in the case of FSEs. CSE **9** with four cinnamoyl moieties and no diisopropylidene bridges showed α-glucosidase inhibition of 74 ± 9%, which is similar to its counterpart CSE **5** with an inhibition value of 77 ± 5%. Interestingly, both CSEs **9** and **5** showed similar α-amylase inhibition values at 99 ± 1% and 98 ± 2%, respectively. This result disagrees with the observations from the FSEs where the inhibition activity decreases in the presence of the diisopropylidene bridges [26]. There was no significant increase in the % inhibition of both enzymes with the increase in the number of cinnamoyl moieties from three to four (compare values for CSEs **5** vs. **9**, and **4** vs. **8**). This observation contrasts with the results observed in the case of FSEs [26]. This is attributed to the lack of hydrogen bonding, which is observed in the case of FSEs from docking studies [26]. In the case of FSEs, hydrogen bonding increased the % inhibition of the enzymes due to stronger interactions with the enzyme moieties [26]. However, the inhibition values of CSEs **5** and **9** with four cinnamoyl moieties, were several folds higher than those of the CSEs **3**, **4**, **6,** and **7** with two of three cinnamoyl moieties, indicating that the number of substituents on the sucrose core plays a key role in enzyme inhibition. CSEs **3**, **4**, **6**, **7**, and **8** showed similar α-glucosidase inhibition values as compared to acarbose at 27 ± 4% (Figure 3). However, CSEs **5** and **9** with four cinnamoyl moieties had almost double the inhibitory values compare to acarbose making them more potent inhibitors. Interestingly, except for CSEs **5** and **9**, the rest of the CSEs had much lower % α-amylase inhibition values compared to α-glucosidase inhibition. This selectivity is important for reducing the gastrointestinal side effects.

The general inhibitory values of the CSEs **2**–**9** increase in the following order, mono-cinnamoyl moieties < di-cinnamoyl ≤ tri-cinnamoyl < tetra-cinnamoyl. The diisopropylidene bridges and acetyl groups have a modest impact on the inhibition values. In many aspects, the investigated CSEs showed similar trends observed in the case of FSEs but also contrasted in some aspects.

The IC_50_ values for CSEs **5** and **9** were calculated for α-glucosidase and α-amylase inhibition. In the case of α-glucosidase, both CSEs **5** and **9** showed similar IC_50_ values of 9 ± 3 μM, which is over 36-fold lower than acarbose at 328 ± 7 μM making them much more potent inhibitors than acarbose (Table 1). In the case of α-amylase, CSEs **5** and **9** showed IC_50_ values of 1 ± 0.2 μM and 0.8 ± 0.1 μM, respectively, which are five-fold less than the value for acarbose 5 ± 0.1 μM (Table 1). Unlike acarbose, which is far more potent inhibitor of α-amylase than α-glucosidase, CSEs **5** and **9** displayed potent inhibition of both the enzymes.

### 2.3. Molecular Docking of CSE ***9*** with α-Glucosidase

**Figure 4:**
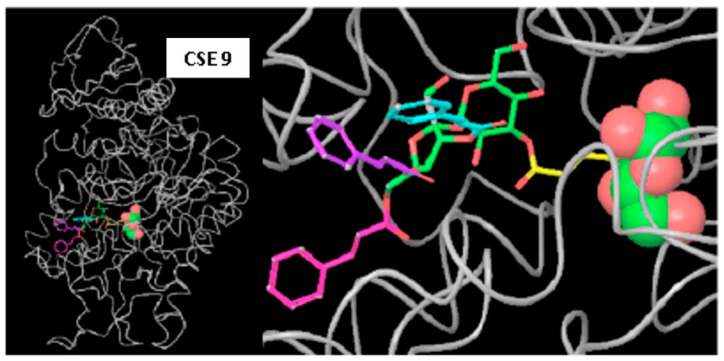
Binding mode picture for CSE **9** docked with α-glucosidase.

**Figure 5:**
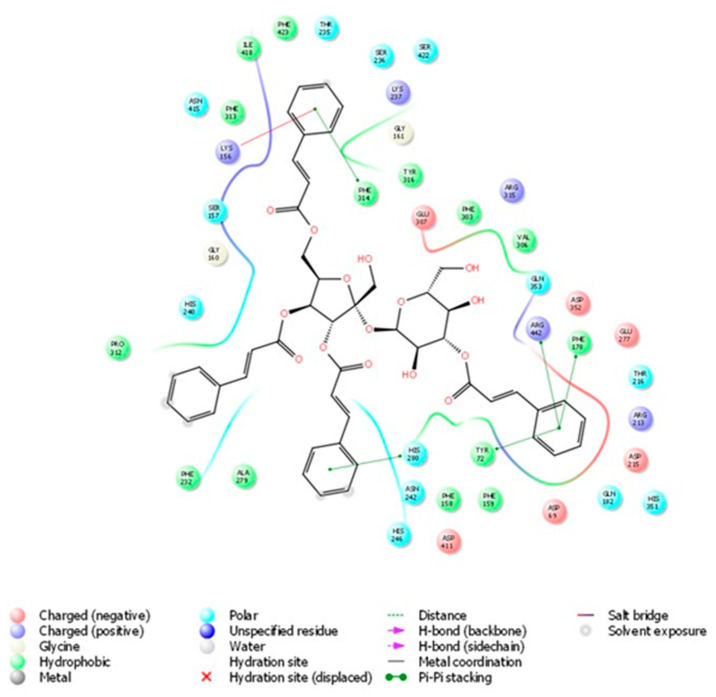
Ligand Interaction Diagram for CSE **9** docked with α-glucosidase.

CSE **9** was docked with α-glucosidase to determine its binding modes and interactions. Since the protein structure of yeast α-glucosidase is unavailable, a homology model was built using yeast isomaltase (PDB ID 3A4A). The site of docking, as well as the amino acid residues that interact with the CSE **9**, are presented in the binding modes and the Ligand Interaction Diagrams (LIDs) (Figure 4 and Figure 5). The cinnamoyl moiety at O-3 has three π-π stacking interactions with the amino acid residues, PHE178, ARG442, and TYR72. This corroborates with the increased inhibition observed in the in vitro results with the incorporation of the cinnamoyl moiety at O-3 (Figure 3). The cinnamoyl moieties at O-3′ and O-6′ interact with HIS280 and PHE314, respectively, through π-π stacking interactions. The cinnamoyl moiety at O-6′ also forms a salt bridge with LYS156 (Figure 4). We hypothesize that the high inhibition value of CSE **9** at 74 ± 9% is attributed to multiple π-π interactions, projection of the cinnamoyl moiety at O-3 (depicted in yellow Figure 4) into the active site of the enzyme, and stabilization of its conformation facilitated by the other moieties at O-3′, O-4′ and O-6′. Unlike the cases of FSEs, though the number of hydrogen bonds that CSE **9** forms is limited due to a lack of aromatic OH groups, π-π interaction, and other hydrophobic interactions make up for this to give high inhibition of α-glucosidase [26].

### 2.4. Molecular Docking of CSEs ***9*** and ***5*** with α-Amylase

**Figure 6:**
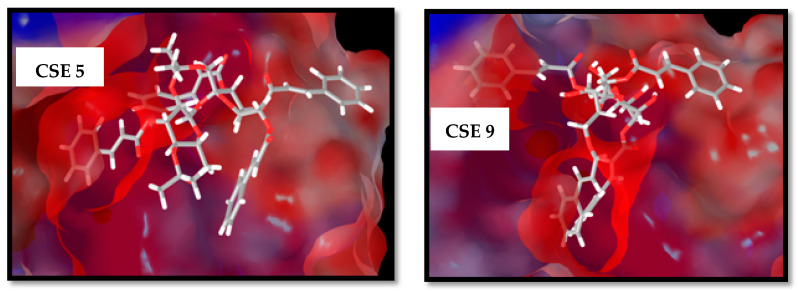
Binding mode pictures for CSEs **5** and **9** docked with α-amylase.

**Figure 7:**
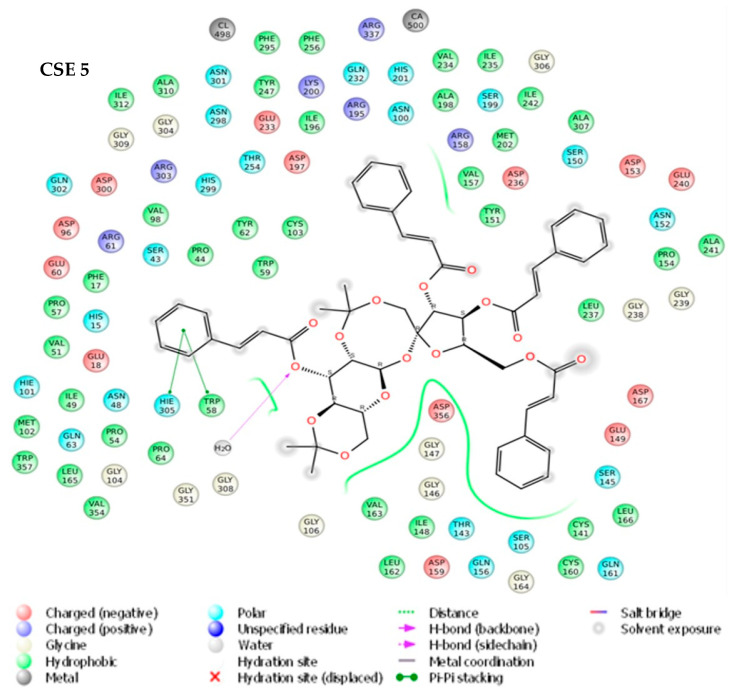

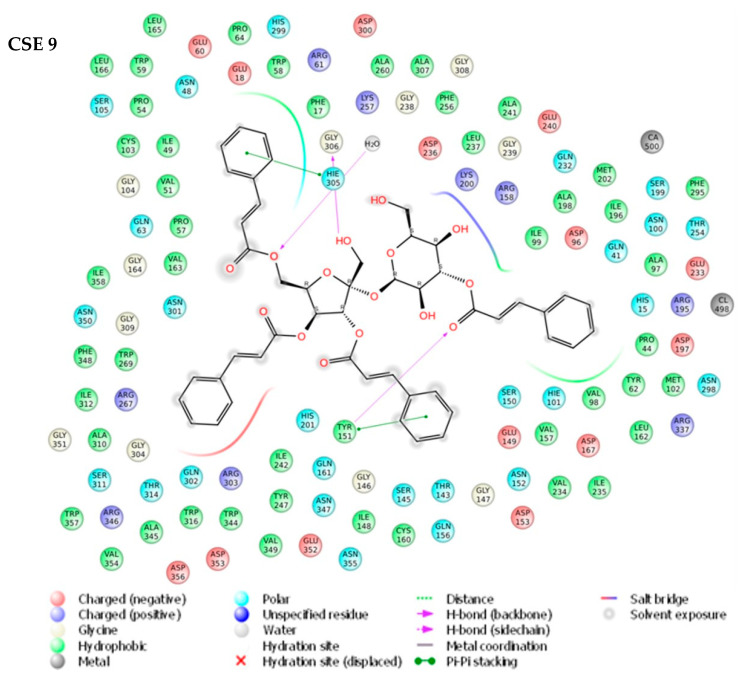
Ligand Interaction Diagrams for CSEs **5** and **9** docked with α-amylase.

CSEs **5** and **9** were chosen since CSE **5** has a restricted configuration due to the bridging of the glucose and fructose moieties by the diisopropylidene rings while CSE **9** is more flexible. Binding mode pictures and the Ligand Interaction diagrams of CSEs **5** and **9** are presented in Figure 6 and Figure 7, respectively. Both CSEs **5** and **9** docked in a similar region around amino acid residues 50–63; 145–151; 197–200; 233–240; and 300–306 (depicted in red in Figure 7). From the LID of CSE **5**, the cinnamoyl moiety at O-3 is involved in π-π stacking interactions with HIE 305 and TRP 58. This highlights the importance of the cinnamoyl at O-3 for inhibition of α-amylase. CSE **9,** which has greater conformational flexibility, has greater interactions with the enzyme. Hence, the cinnamoyl moiety at O-3 forms a hydrogen bond with TYR 151, and the cinnamoyl at O-3′ shows π-π stacking with TYR 151. The cinnamoyl moiety at O-6′ shows π-π stacking with HIE 305, and OH-2′ forms a hydrogen bond with GLY 306. The high inhibition of CSEs **5** and **9** could be attributed to multiple π-π interactions and favorable projection of the cinnamoyl moiety at O-3 in the active site of the enzyme. It is noted that these compounds show similar interactions to the FSEs except they lack phenolic OH bonding with the residues.

## 3. Methods

### 3.1. Chemicals

All the chemicals and reagents used in this study were purchased from Sigma Aldrich (α-glucosidase, α-amylase from porcine pancreas, acarbose, 4-nitrophenyl α-d-glucopyranoside (PNPG), Dinitro Salicylic Acid (DNSA), DMSO, NaHPO_4_, Na_2_HPO_4_).

### 3.2. In Vitro α-Glucosidase Inhibition Assay

α-Glucosidase inhibition was tested according to previously reported methods with some modifications [28]. 8 µL of CSEs or control (Acarbose) in DMSO were added to 115 µL of 0.1 M sodium phosphate buffer pH 7.0 in a 96 well microtiter plate (50 µg/mL final concentration). Fifty microliters of yeast α-glucosidase enzyme solution (0.5 U/mL in phosphate buffer) was added to the wells. The plate was incubated at 37 °C for 15 min. Next, 25 µL of 2.5 mM PNPG (4-nitrophenyl α-d-glucopyranoside) substrate solution (in phosphate buffer) was added. The microplate was incubated at 37 °C for another 15 min. The absorbance was then measured using a microplate reader at 405 nm. The percentage inhibition was calculated using the formula:

$$\%\;inhibition\;=\;\frac{\Delta Abs_{control}-\Delta Abs_{sample}}{\Delta Abs_{control}}\;\times \;100$$

### 3.3. In Vitro α-Amylase Inhibition Assay

α-amylase inhibition was tested using previously reported methods [11] 50 µL of the CSEs or control (Acarbose) in DMSO (final concentration 50 µg/mL) were taken in test tubes to which 100 µL of porcine pancreatic α-amylase (5 U/mL) and 460 µL of 0.05 M sodium phosphate buffer pH 6.8 were added. The tubes were then incubated at 37 °C for 10 min. Next, 450 µL of 0.5% starch solution was added and the tubes were incubated for a further 20 min at 37 °C. Finally, 500 µL of Dinitro Salicylic Acid (DNSA) reagent was added and the tubes were placed in a boiling water bath for 15 min. The absorbance of the tubes was recorded at 540 nm and the % inhibition calculated using the same formula as for α-glucosidase,

$$\%\;inhibition\;=\;\frac{\Delta Abs_{control}-\Delta Abs_{sample}}{\Delta Abs_{control}}\;\times \;100$$

### 3.4. Calculation of IC_50_ and Statistical Analysis for Enzyme Inhibition

All measurements were performed in triplicates and the values. The IC_50_ values for selected CSEs were calculated by plotting a dose-response curve for a range of concentrations of the CSEs (0–25 µg/mL) for the selected CSEs (data not shown) and regression analysis was done using Graphpad Prism software using the in-built function.

### 3.5. Molecular Docking of the CSE ***9*** with α-Glucosidase

A suitable template for building a homology model for α-glucosidase was found using the ‘BLAST’ algorithm. Yeast isomaltase (PDB ID: 3A4A) *(Saccharomyces cerevisiae)* which shared 72% sequence identity and 85% similarity with yeast α-glucosidase was used as a template to construct the homology model [11]. AutoDock was used for docking simulations and Maestro was used to visualize the binding modes. Receptor preparation was done by first removing water molecules and then adding Gasteiger charges and hydrogen atoms. Maximum number of rotatable bonds was chosen to increase the flexibility of the docked ligand. The site that houses the glucose molecule in the yeast isomaltase was chosen to be the gridded binding site. Lamarckian Genetic algorithm was chosen for the docking and a total of 100 binding modes were chosen.

### 3.6. Molecular Docking of the CSEs ***5*** and ***9*** with α-Amylase

The molecular interaction of the CSEs with porcine pancreatic α-amylase was studied using Schrödinger’s Maestro software. The enzyme porcine pancreatic α-amylase (PDB ID: 1OSE) was imported and the structure processed using the Protein Preparation Wizard module. The receptor grid was generated based on the coordinates of the bound acarbose in the crystal structure, known as the primary binding site [11]. The Glide suite was used to perform the docking of the CSEs with the Extra Precision (XP) option. Water molecules that are 5 Å away from the primary binding site were retained during the docking. Residues that possess bonds containing hydroxyl and thiol groups as well as single bonds not having a specific chirality in the ligands were considered to be flexible.

## 4. Conclusions

Cinnamoyl sucrose esters inhibited α-glucosidase and α-amylase to different extents and the inhibition activity depended on the structure of these compounds. Selective inhibition of α-glucosidase over α-amylase to eliminate the side effects is achievable by tuning the number of cinnamoyl moieties, their positions, diisopropylidene bridges, and the acetyl groups. Among the eight compounds tested, CSEs **5** and **9**, with four cinnamoyl moieties, showed much higher inhibition of α-glucosidase than acarbose and near complete inhibition of α-amylase. Both showed much higher IC_50_ values than acarbose. From the in silico docking studies, the high inhibition of CSEs **5** and **9** is thought to be due to multiple π-π interactions and favorable projection of the cinnamoyl moieties (especially O-3 cinnamoyl) in the enzyme pockets. This work demonstrates the potential of CSEs as AGIs. Further studies on developing phenylpropanoid sucrose esters as AGIs with minimum side effects are underway in our lab.

## Figures and Tables

**Table 1:** IC_50_^a^ values for α-glucosidase and α-amylase inhibition by CSEs **5** and **9.**

Entry	CSE	IC_50_ Value (µM)	IC_50_ Value (µM)
		α-Glucosidase	α-Amylase
1	5	9 ± 3	1 ± 0.2
2	9	9 ± 3	0.8 ± 0.1
3	Acarbose	328 ± 7	5 ± 0.1

^a^ IC_50_ values are expressed as means ± SD; *n* = 3; IC_50_ values were calculated using a dose-response curve in GraphPad PRISM software.
